# Gas Sensing Performances of ZnO Hierarchical Structures for Detecting Dissolved Gases in Transformer Oil: A Mini Review

**DOI:** 10.3389/fchem.2018.00508

**Published:** 2018-10-22

**Authors:** He Zhang, Wei-Gen Chen, Yan-Qiong Li, Zi-Hao Song

**Affiliations:** ^1^State Key Laboratory of Power Transmission Equipment & System Security and New Technology, School of Electrical Engineering, Chongqing University, Chongqing, China; ^2^School of Electronic and Electrical Engineering, Chongqing University of Arts and Sciences, Chongqing, China

**Keywords:** ZnO, gas sensors, hierarchical structures, sensitivity, selectivity, stability, gas sensing mechanism

## Abstract

Power transformer is one of the critical and expensive apparatus in high voltage power system. Hence, using highly efficient gas sensors to real-time monitor the fault characteristic gases dissolved in transformer oil is in pressing need to ensure the smooth functionalization of the power system. Till date, as a semiconductor metal oxide, zinc oxide (ZnO) is considered as the promising resistive-type gas sensing material. However, the elevated operating temperature, slow response, poor selectivity and stability limit its extensive applications in the field of dissolved gases monitoring. In this respect, rigorous efforts have been made to offset the above-mentioned shortcomings by multiple strategies. In this review, we first introduce the various ZnO hierarchical structures which possess high surface areas and less aggregation, as well as their corresponding gas sensing performances. Then, the primary parameters (sensitivity, selectivity and stability) which affect the performances of ZnO hierarchical structures based gas sensors are discussed in detail. Much more attention is particularly paid to the improvement strategies of enhancing these parameters, mainly including surface modification, additive doping and ultraviolet (UV) light activation. We finally review gas sensing mechanism of ZnO hierarchical structure based gas sensor. Such a detailed study may open up an avenue to fabricate sensor which achieve high sensitivity, good selectivity and long-term stability, making it a promising candidate for transformer oil monitor.

## Introduction

Power transformer is one of the most critical and expensive devices in high voltage power system (Christina et al., 2018). Generally, oil is used inside the transformer for its operation and can release different fault characteristic gases, such as hydrogen (H_2_), carbon oxides (CO, CO_2_) and hydrocarbons (CH_4_, C_2_H_2_, C_2_H_4_, and C_2_H_6_). Hence, real-time detection of dissolved gases in transformer oil is very essential in order to avoid unexpected failures (Mariprasath and Kirubakaran, 2018). At present, dissolved gas analysis (DGA) remains to be the simplest and most effective diagnostic method for checking latent faults of oil-immersed power transformers (Siada and Hmood, 2015; Fan et al., 2018). Therefore, using highly efficient gas sensors to real-time monitor these dissolved gases in transformer oil is a feasible way to ensure the stability and reliability of power system (Uddin et al., 2016).

Different types of gas sensors have already been applied in the online detection of dissolved gases in transformer oil, such as resistance-type (Benounis et al., 2008; Sun et al., 2015), optical-type (Ma et al., 2012) and electrochemical-type (Ding et al., 2014). Among diverse types of gas sensors, resistance-based sensors stand out owing to advantages like the small, cheap, high sensitivity and low power consumption (Bodzenta et al., 2002; Yang et al., 2011; Zhao et al., 2017; Xu et al., 2018). With the increasing demand for better gas sensors of higher sensitivity and selectivity (Sun et al., 2012; Gardon and Guilemany, 2013), countless endeavors have been poured on hunting for more suitable sensing nanomaterials. Semiconductor metal oxides (MOS), such as zinc oxide (ZnO), tin oxide (SnO_2_), tungsten oxide (WO_3_), etc., have received wide research for gas sensing applications and so on. Among these, the gas sensing performance of ZnO-based gas sensor was first investigated by Seiyama et al. (1962). As a typical n-type semiconductor material with a direct wide band gap (Eg ≈ 3.37 ev) and large excitation binding energy (~60 mev), ZnO has got important status in various MOS nanomaterials due to its high carrier mobility of conduction electrons, good chemical and thermal stability (Zeng et al., 2015; Das and Sarkar, 2017; Ganesh et al., 2017).

The gas sensing properties of ZnO greatly depend on its structure and morphology including surface area, size, orientation and crystal density (Cho et al., 2011). Therefore, tailoring the structure and morphology of ZnO is particularly important to optimize the gas sensing performances (Liao et al., 2008). In particular, the elaborate design of unique three-dimensional (3D) hierarchical architectures can fully achieve this, since such hierarchical structures possess high surface area and fast gas diffusion as well as reduce the agglomerated configuration of low dimensional structures.

ZnO-based gas sensors commonly have the shortcomings of slow response, poor selectivity and lack of long-term stability, which limits the wide applications. To acquire an efficient and reliable dissolved gases sensor, high sensitivity, selectivity, long-term stability, low response / recovery time, low fabrication cost are urgently needed (Wang et al., 2012). This review focuses on the factors that affect the performances (sensitivity, selectivity and stability), the methods to improve these sensor parameters and gas sensing mechanism of ZnO-based gas sensors.

## Gas sensing performances of ZnO-based gas sensor

### Effects of morphologies about ZnO hierarchical structures on gas sensing performances

**Figure 1:**
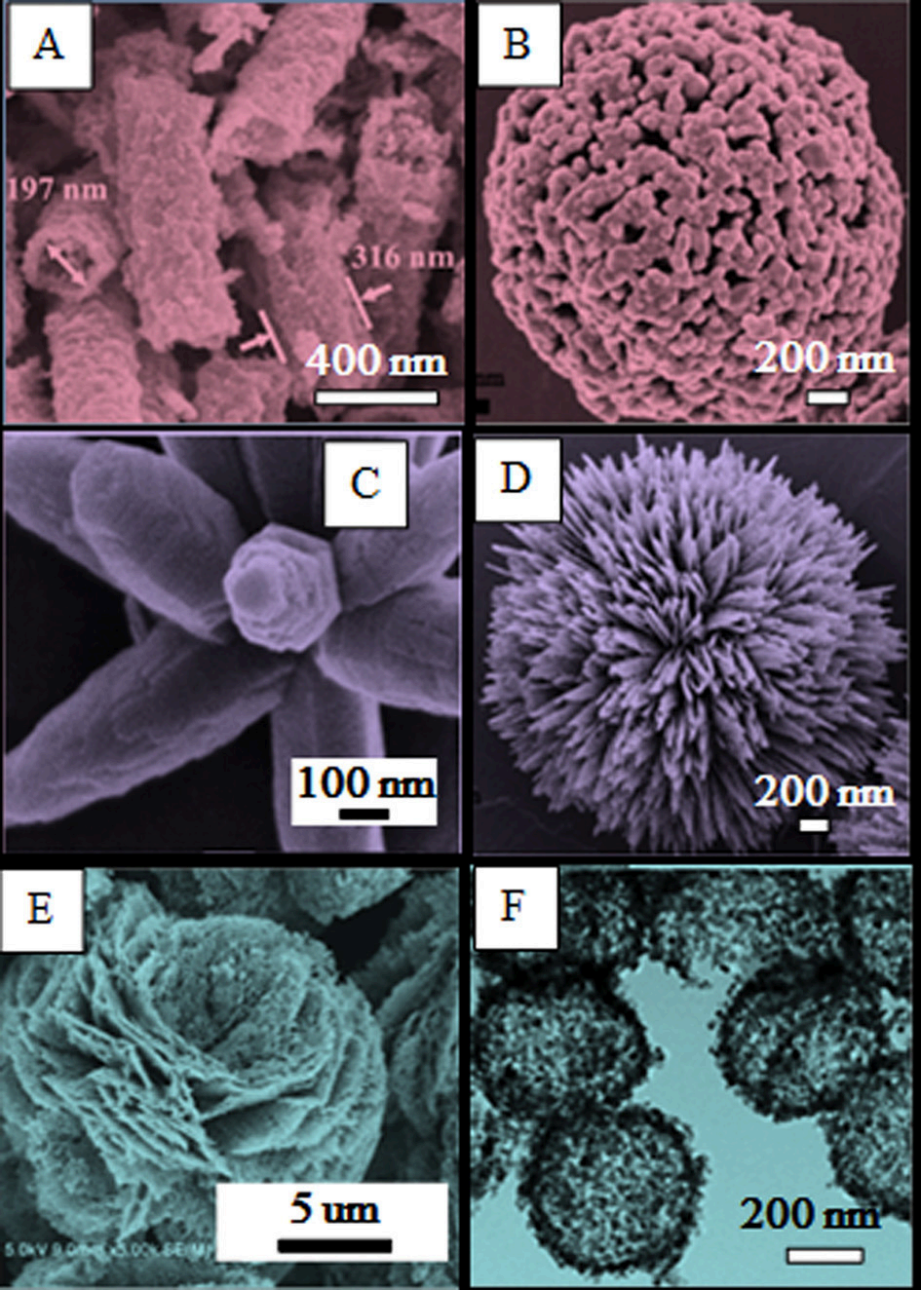
ZnO with different 3D hierarchical structures: **(A)** Nanofibers assembly by 0D structures. Reprinted with permission from Li X. et al. (2015). Copyright (2015) Elsevier Science BV. **(B)** Nanospheres assembly by 0D structures. Reprinted with permission from Chen H. et al. (2016). Copyright (2016) Elsevier Science SA. **(C)** Flower-like microstructure assembly by 1D structures. Reprinted with permission from Lin et al. (2015). Copyright (2015) Elsevier Science SA. **(D)** Sea-urchin-like ZnO nanostructures assembly by 1D structures. Reprinted with permission from Chen H. et al. (2016). Copyright (2016) Elsevier Science SA. **(E)** Porous flower-like ZnO nanostructures assembly by 2D structures. Reprinted with permission from Gu et al. (2011). Copyright (2011) Elsevier Science SA. **(F)** Core-shell hollow spheres assembly by 2D structures. Reprinted with permission from Han et al. (2016). Copyright (2016) Elsevier Science SA.

Three-dimensional (3D) hierarchical structures are generally recognized as the best candidate for gas sensing performances, compared with low-dimensional structures (Mo et al., 2008; Guo, 2016). They are defined as those assembled by zero-dimensional (0D), one-dimensional (1D) and two-dimensional (2D) components, which can be further classified into the following sub-sections. (1) Assembly of 0D structures: Li W. Q. et al. (2015) reported the synthesis of pure ZnO hollow nanofibers by electrospinning method. The walls of ZnO nanofibers consist of the aggregation of many individual nanoparticles, as shown in Figure 1A. The sensor based on ZnO hollow nanofibers exhibits excellent sensing performance for acetone detection, which can be attributed to the large aperture and small diameters provide higher specific surface area for gas adsorption. Chen H. et al. (2016) synthesized the uniformly monodispersed ZnO nanospheres via a simply hydrothermal route. In particular, all the microparticles on the surface are sphere-shapes and have a rough surface, as shown in Figure 1B. This unique porous structure exhibits perfect sensing performance toward ethanol. (2) Assembly of 1D structures: Lin et al. (2015) reported the hierarchical ZnO microstructures by hydrothermal method. The morphology of the sample likes a bunch of flowers which is made of uniform nanorods, as shown in Figure 1C. The sensor based on the sample shows a good response. Chen H. et al. (2016) reported the sea-urchin-like ZnO nanostructures by hydrothermal method. The sample is composed of many strips and radiates from the center, as shown in Figure 1D. The sensor based on the ZnO sample toward ethanol exhibits high sensitivity and quick response. (3) Assembly of 2D structures: Gu et al. (2011) reported the porous flower-like ZnO nanostructures by economical hydrothermal synthesis combined with subsequent calcination. Calcination of the precursors produced flower-like ZnO nanostructures which composed of interconnected porous ZnO nanosheets with high porosity, as shown in Figure 1E. The as-prepared flower-like ZnO nanostructures are highly promising candidate for applications of gas sensors. Han et al. (2016) reported the ZnO hollow spheres with high crystallinity via a simple template process, as shown in Figure 1F. The surfaces of these core-shell spheres are rough, suggesting that polystyrene sphere (PSS) core was coated by ZnO nanoparticles. The sensor based on ZnO hollow spheres exhibits good sensing performances.

In this part, the authors make a brief introduction with respect to the hierarchical structures. Hierarchical hollow or porous ZnO structures exhibit excellent properties for gas sensor applications (Guo et al., 2011, 2012, 2013). These unique hollow structures with large specific surface area and highly porous structures can provide excellent channel and “surface accessibility” for the gas transportation, which is very favorable for facilitating the interaction of ZnO surface with the gas molecules (Gu et al., 2011). No matter how complicated the hierarchical structure, it's all derived from low dimensional nanostructures as building blocks. Hence, the investigation about regulating the structure and morphology is a meaningful and challenge work.

A summary about factors affecting gas sensing performances of ZnO-based gas sensors and improvement approaches is shown in Table 1. The details are described in sections Factors affecting the sensitivity of ZnO hierarchical structure based Gas sensor, Factors affecting the selectivity of ZnO hierarchical structure based Gas sensor, and Factors affecting the long-term stability of ZnO hierarchical structure based Gas sensor as follows.

**Table 1:** A summary about factors affecting gas sensing performances of ZnO hierarchical structure based gas sensors and improvement approaches.

**Main characteristic indexes which reflect the performances**	**Influencing factors and improvement approaches**	**References**
Sensitivity	Modulation of the dimensional and the exposed crystal facet of their constituting building blocks	Zhang et al., 2009
	Enhance the porosity of hierarchical structures	Lei et al., 2017; Song et al., 2018
	Modification by doping with noble metals and loading other n-type or p-type MOS materials	Lin et al., 2015
	Control of grain size	Mirzaei et al., 2018
Selectivity	Dope with noble metals and p-type metal oxides	Li T. M. et al., 2015
	Lower the operating temperature by activating the sensing material under UV illumination	Chen Y. et al., 2016; Espid and Taghipour, 2017
Long-term stability	Calcination/annealing as the post-processing treatment	Gu et al., 2011
	Reduce the working temperature of gas sensing element	Chen Y. et al., 2016
	Dope noble metal or synthesis of mixed oxides	Dey, 2018

### Factors affecting the sensitivity of ZnO hierarchical structure based gas sensor

Recently, numerous reports confirmed that ZnO-based nanomaterials are promising candidates for the fabrication of gas sensors (Gu et al., 2013; Wang et al., 2014). Given this, a number of strategies have been proposed for enhancing the gas sensitivity. It can be introduced from the following four aspects.

- Modulation of the dimensional and the exposed crystal facet of their constituting building blocks.

Zhang et al. (2009) synthesized brush-like hierarchical ZnO nanostructures. The FESEM image (Supplementary Figure 1A) shows that this structure is composed of 6-fold nanorod arrays grown on the side surface of core nanowires. The central stems provide its six prismatic facets as growth platforms for branching of multipod units. The sensor based on these structures shows high sensitivity and fast response.

- Enhance the porosity of hierarchical structures.

Lei et al. (2017) successfully synthesized hierarchical porous ZnO microspheres assembled from 2D nanosheets. The high specific surface area and hierarchical pore structure are beneficial to increase the adsorption capacity (Supplementary Figure 1B). Song et al. (2018) reported hierarchical porous ZnO microflowers which composed of ultrathin nanosheets. From the SEM image (Supplementary Figure 1C), we can see that the surface of nanosheet has lots of pores. The porous structure is favorable for gas sensor to promote the inward/outward gas diffusion and improve gas sensitivity.

- Modification by doping with noble metals and loading other n-type or p-type MOS materials.

It's known that noble metals, such as Pt (Rout et al., 2006), Pd (Yang et al., 2010) and Au (Vallejos et al., 2011) are frequently used in gas sensing materials due to doping can sensitize the ZnO electronic and structural properties. Lin et al. (2015) reported that Au nanoparticles were decorated on the surface of hierarchical flower-like ZnO microstructures, as shown in Supplementary Figure 1D. After Au nanoparticle-decoration, the specific surface area is much higher than that of the bare ZnO (Figure 1C). Au nanoparticles can act as catalysts to accelerate the chemisorption process and greatly improve the sensitivity. So far, heterostructure composites consisting of two metal oxides, such as (n-n type) SnO_2_/ZnO (Park et al., 2013) and (n-p type) NiO/ZnO, AgO/ZnO (Gandomania et al., 2014) have been successfully prepared and have improved the sensitivity. Liu et al. (2017) reported the NiO nanoparticles which were decorated onto the surfaces of well-dispersed ZnO hollow spheres (Supplementary Figures 1E,F). Such hollow structures with rough surfaces endow the NiO/ ZnO composites high surface areas and abundant active sites, which could facilitate the gas diffusion toward the entire materials and an improvement of the sensitivity (Lee, 2009).

- Control of grain size.

Previous research found that sensors which consist of fine particles of MOS tend to exhibit high sensitivity. Thus, one of the most important factors affecting the sensitivity is grain size (D) of the sensor materials in conjunction with the thickness of the space charge layer (L). Supplementary Figure 1G illustrates three kinds of schematic models for grain-size effects (Shimizu and Egashira, 1999). When D >> 2L, the conductance is limited by Schottky barrier at grain boundaries (grain boundary control). If D ≥ 2L, the conductance is limited by necks between grains (neck control). When D < 2L, the conductance is controlled by grains themselves (grain control). Among three models, grain control is the most sensitive condition (Mirzaei et al., 2018). The smaller the grain size, the higher the sensitivity of gas sensor. But, excessive decrease in grain size can reduce structural stability.

### Factors affecting the selectivity of ZnO hierarchical structure based gas sensor

Selectivity is the ability of gas sensor to recognize the target gas in a mixture of other gases. Generally, there are two approaches for enhancing the selectivity of gas sensor. The first one is to synthesize a material which is selective to the specific compound and has very low cross-sensitivity for other compounds. Moreover, the synergistic effect of two component system is greater than the production effect of the two elements. In fact, noble metals and p-type metal oxides have been extensively applied as good catalysts in the two component systems to promote selectivity of sensors (Li T. M. et al., 2015). Another approach to improve the selectivity is to combine with other methods. Recently, some reports have suggested that lowering the operating temperature can be realized by activating the sensing material under UV illumination (Helwig et al., 2009; Lu et al., 2012; Cui et al., 2015). The possible UV-activated selective photo catalysis plays an important role in the enhancement of the selectivity at low temperature (Li X. et al., 2015). It can be explained based on the selective photocatalytic oxidation. The adsorbed oxygen would be re-activated by the photon generated electron-hole pairs, which is conductive to enhancing their reactivity with target gas. After the target gas reacted with the adsorbed oxygen on ZnO surface, the donated electrons would thus decrease resistance of the sensor and finally reduce the operating temperature (Ho et al., 2015). Chen et al. reported that the mesoporous hollow ZnO microspheres were applied to detect volatile organic vapors (VOCs) with the help of UV LED illumination at lower temperatures (Chen Y. et al., 2016). The sensor with UV activation at 80°C shows a much higher response to ethanol (Supplementary Figure 2A). When the sensor was operated at 220°C, the UV illumination became ineffective. It shows almost same response to ethanol and acetone (Supplementary Figure 2B). This is because the difference about catalytic conversion of O^2−^ would have negligible toward them at 220°C. However, the O^−^ possibly indicated higher preference to ethanol at 80°C, resulting in the better selectivity. When metal doped-ZnO was illuminated by UV light, the sensor had an appreciable selectivity at low temperature, which was attributed to the heterostructure was in favor of chemical interactions, adsorption of gases and changes in electronic bind energies in the composite (Chen et al., 2008). Espid investigated the photo-responsive behavior of ZnO/ In_2_O_3_ composite sensors (Espid and Taghipour, 2017). When the semiconductor composites are irradiated with photons emitted from a UV source, the photo-generated electron/hole pairs will enhance the conductance of the sensing layer and improve the selectivity.

### Factors affecting the long-term stability of ZnO hierarchical structure based gas sensor

Stability is a key parameter for the long-term development of gas sensors, which determines its application state in the real market. Generally, the long-term stability refers to the attenuation degree of gas sensing performances (e.g., sensitivity, selectivity, response and recovery time) during a certain period of time. When the sensor is in working state, working conditions including high temperature and toxic gases can reduce the stability. When the sensor is in normal storage state, changes of humidity, fluctuations of temperature in the surrounding atmosphere may also interfere with the stability of sensor. At present, there is not a recognized method to improve stability of ZnO-based gas sensors. Stability can be increased to some extent by calcination/ annealing as the post-processing treatment (Gu et al., 2011) and reducing the working temperature of gas sensing element. Chen et al. tested the long-term stability of ZnO-based sensor working at 80°C with UV activitation (Chen Y. et al., 2016). The sensor test lasted 1 month (Supplementary Figure 2C). In the first 2 days, the response values dropped significantly, which might be related to the “pre-aging” effect. In the next few days, the sensor response became stabilized and showed a good long-term stability. It might be because the microstructure of the materials had little change under low temperature with low-powered UV activation. In addition, doping noble metal or synthesis of mixed oxides can also increase the stability of the sensors (Dey, 2018).

## Gas sensing mechanism of ZnO hierarchical structure based gas sensor

By summarizing the methods to improve the gas sensing performances in section Gas Sensing Performances of ZnO-Based Gas Sensor, we find that metal doping is an excellent method to promote sufficient reaction between sensing material and target gas.

The gas sensing mechanism of noble metals doped-ZnO hierarchical structures based gas sensors is explained as an example. This process mainly involves two effects: chemical effect and electronic effect (Zhu and Zeng, 2017). Firstly, the chemical effect is related to spillover process (Nakate et al., 2016). Oxygen molecules were adsorbed on the surface and grain boundary of ZnO, forming the oxygen ions. The sensitization of noble metals increases the quantity of oxygen species and accelerates the surface reaction, causing an expansion of charge depletion layer, which results in a higher baseline resistance (Supplementary Figures 3A,B). When the reducing gas is introduced, the catalysis of noble metals may give rise to the dissociation of target gas molecules. The trapped electrons are released and transmitted to the conduction band, resulting in a remarkable decrease in depletion layer with a lower resistance. Secondly, the electric effect is produced by contact resistance of noble metal modified ZnO gas sensors (Hosseini et al., 2015). Electrons from the conduction band of ZnO transfer into noble metals owing to their work functions are different, forming the Schottky barriers at noble metal-ZnO interface, which leading to generate the additional depletion region near ZnO surface (Supplementary Figure 3C).

Therefore, the enhanced sensing performance was ascribed to the spillover phenomenon, the formation of Schottky barriers at the interface between noble metals and ZnO, more introduced surface active sites and effective surface areas (Hosseini et al., 2015).

## Conclusion

A study on gas sensing performances of ZnO hierarchical structures has been shortly summarized in this review. Firstly, unique 3D hierarchical architectures with high sensing capabilities are discussed by modifying surface morphologies. Small grain size, high effective specific surface area and porosity are favorable to the enhancement of gas sensing performances. Therefore, the preparation of the desired 3D hierarchical structure can lay a solid foundation for the development of gas sensor. Then, factors that affect the sensitivity, selectivity and stability of ZnO hierarchical structures based gas sensors and their improvement strategies are summarized separately. Among these methods, additive doping and UV-light irradiation are more effective methods to improve gas sensing performances. The former can increase charge carrier concentration and decrease activation energy. The latter can promote the catalytic oxidation reaction between target gases and oxygen ions, thus reduce the working temperature and power consumption. Numerous reports indicate that the integration of metal doped-oxide and UV excitation is one of the most effective and workable attempts to achieve high sensor performances. The composite oxides based sensors under UV illumination have better charge separation, which benefit for the gas performances enhancement of the sensors. We hope our work is helpful for further exploration on higher gas sensing performances of MOS sensing materials for detecting dissolved fault gases in transformer oil. Finally, gas sensing mechanism of noble metal sensitized ZnO is illuminated from the point of view of chemical effect and electronic effect. Nevertheless, the authors suggest only a few possible ways to improve the existed oxygen-absorbed model in recent researchers. Much effort should be made to hunt for an integration of different models which was used to explain the gas sensing reaction.

## Author contributions

HZ and W-GC conceived and designed the experiments, HZ and Y-QL performed the experiments, HZ and Z-HS analyzed the data, HZ wrote the manuscript with input from all authors. All authors read and approved the manuscript.

### Conflict of interest statement

The authors declare that the research was conducted in the absence of any commercial or financial relationships that could be construed as a potential conflict of interest.

## References

[B1] Benounis T. M. Ngnui T. A. Jaffrezic N. Dutasta J. P. (2008). NIR and optical fiber sensor for gases detection produced by transformation oil degradation. Sens. Actuators A Phys. 141, 76–83. 10.1016/j.sna.2007.07.036

[B2] Bodzenta J. Burak B. Gacek Z. Jakubik W. P. Kochowski S. Urbanczyk M. (2002). Thin palladium film as a sensor of hydrogen gas dissolved in transformer oil. Sens. Actuators B Chem. 87, 82–87. 10.1016/S0925-4005(02)00221-6

[B3] Chen H. Ma S. Y. Jiao H. Y. Yang G. J. Xu X. L. Wang T. T. (2016). The effect microstructure on the gas properties of Ag doped zinc oxide sensors: spheres and sea-urchin-like nanostructures. J. Alloy Compd. 687, 342–351. 10.1016/j.jallcom.2016.06.153

[B4] Chen L. Y. Bai S. L. Zhou G. J. Li D. Q. Chen A. F. Chung C. L. (2008). Synthesis of ZnO-SnO_2_ nanocomposites by microemulsion and sensing properties for NO_2_. Sens. Actuators B Chem. 134, 360–366. 10.1016/j.snb.2008.04.040

[B5] Chen Y. Li X. G. Li X. X. Wang J. Tang Z. N. (2016). UV activated hollow ZnO microspheres for selective ethanol sensors at low temperatures. Sens. Actuators B Chem. 232, 158–164. 10.1016/j.snb.2016.03.138

[B6] Cho S. Kim S. Jung D. W. Lee K. H. (2011). Formation of quasi-single crystalline porous ZnO nanostructures with a single large cavity. Nanoscale 3, 3841–3848. 10.1039/c1nr10609k 21842089

[B7] Christina A. J. Salam M. A. Rahman Q. M. Wen F. S. Ang S. P. Voon W. (2018). Causes of transformer failures and diagnostic methods: a review. Renew. Sust. Energ. Rev. 82, 1442–1456. 10.1016/j.rser.2017.05.165

[B8] Cui J. B. Shi L. Q. Xie T. F. Wang D. J. Lin Y. H. (2015). UV-light illumination room temperature HCHO gas-sensing mechanism of ZnO with different nanostructures. Sens. Actuators B Chem. 227, 220–226. 10.1016/j.snb.2015.12.010

[B9] Das M. Sarkar D. (2017). One-pot synthesis of zinc oxide-polyaniline nanocomposite for fabrication of efficient room temperature ammonia gas sensor. Ceram. Int. 43, 11123–11131. 10.1016/j.ceramint.2017.05.159

[B10] Dey A. (2018). Semiconductor metal oxide gas sensors: a review. Mater. Sci. Eng. B 229, 206–217. 10.1016/j.mseb.2017.12.036

[B11] Ding J. Li X. Cao J. Sheng L. Yin L. Xu X. (2014). New sensor for gases dissolved in transformer oil based on solid oxide fuel cell. Sens. Actuators B Chem. 202, 232–239. 10.1016/j.snb.2014.05.061

[B12] Espid E. Taghipour F. (2017). Development of highly sensitive ZnO/In_2_O_3_ composite gas sensor activated by UV-LED. Sens. Actuators B Chem. 241, 828–839. 10.1016/j.snb.2016.10.129

[B13] Fan J. M. Wang F. Sun Q. Q. Ye H. S. Jiang Q. J. (2018). Application of polycrystalline SnO_2_ sensor chromatographic system to detect dissolved gases in transformer oil. Sens. Actuators B Chem. 267, 636–646. 10.1016/j.snb.2018.04.014

[B14] Gandomania S. K. Yousefib R. Sheinic F. J. Huang N. M. (2014). Optical and electrical properties of p-type Ag-doped ZnO nanostructures. Ceram. Int. 40, 7957–7963. 10.1016/j.ceramint.2013.12.145

[B15] Ganesh R. S. Durgadevi E. Navaneethan M. Patil V. L. Ponnusamy S. Muthamizhchelvan C. (2017). Low temperature ammonia gas sensor based on Mn-doped ZnO nanoparticle decorated microspheres. J. Alloys Compd. 721, 182–190. 10.1016/j.jallcom.2017.05.315

[B16] Gardon M. Guilemany J. M. (2013). A review on fabrication, sensing mechanisms and performance of metal oxide gas sensors. J. Mater. Sci. Mater. Electron. 24, 1410–1421. 10.1007/s10854-012-0974-4

[B17] Gu C. P. Huang J. R. Wu Y. J. Zhai M. H. Sun Y. F. Liu J. H. (2011). Preparation of porous flower-like ZnO nanostructures and their gas-sensing property. J. Alloy Compd. 509, 4499–4504. 10.1016/j.jallcom.2010.11.078

[B18] Gu C. P. Li S. S. Huang J. R. Shi C. C. Liu J. H. (2013). Preferential growth of long ZnO nanowires and its application in gas sensor. Sens. Actuators B Chem. 177, 453–459. 10.1016/j.snb.2012.11.044

[B19] Guo W. W. (2016). ZnO nanosheets assembled different hierarchical structures and their gas sensing properties. J Mater Sci. Mater. Electron. 27, 7302–7310. 10.1007/s10854-016-4699-7

[B20] Guo W. W. Liu T. M. Sun R. Chen Y. Zeng W. Wang Z. C. (2013). Hollow, porous, and yttrium functionalized ZnO nanospheres with enhanced gas-sensing performances. Sens. Actuators B Chem. 178, 53–62. 10.1016/j.snb.2012.12.073

[B21] Guo W. W. Liu T. M. Zeng W. Liu D. J. Chen Y. Wang Z. C. (2011). Gas-sensing property improvement of ZnO by hierarchical flower-like architectures. Mater. Lett. 65, 3384–3387. 10.1016/j.matlet.2011.07.059

[B22] Guo W. W. Liu T. M. Zhang H. J. Sun R. Chen Y. Zeng W. (2012). Gas-sensing performance enhancement in ZnO nanostructures by hierarchical morphology. Sens. Actuators B Chem. 166, 492–499. 10.1016/j.snb.2012.02.093

[B23] Han B. Q. Liu X. Xing X. X. Chen N. Xiao X. C. Liu S. Y. (2016). A high response butanol gas sensor based on ZnO hollow spheres. Sens. Actuators B Chem. 237, 423–430. 10.1016/j.snb.2016.06.117

[B24] Helwig A. Müller G. Sberveglieri G. Eickhoff M. (2009). On the low- temperature response of semiconductor gas sensors. J. Sens. 2009, 1–17. 10.1155/2009/620720

[B25] Ho Y. H. Huang W. S. Chang H. C. Wei P. K. Sheen H. J. Tian W. C. (2015). Ultraviolet-enhanced room-temperature gas sensing by using floccule-like zinc oxide nanostructures. Appl. Phys. Lett. 106, 1831031–1831034. 10.1063/1.4919921

[B26] Hosseini Z. S. Mortezaali A. Iraji A. Fardindoost S. (2015). Sensitive and selective room temperature H_2_S gas sensor based on Au sensitized vertical ZnO nanorods with flower-like structures. J. Alloys Compd. 628, 222–229. 10.1016/j.jallcom.2014.12.163

[B27] Lee J. H. (2009). Gas sensors using hierarchical and hollow oxide nanostructures: overview. Sens. Actuators B Chem. 140, 319–336. 10.1016/j.snb.2009.04.026

[B28] Lei C. S. Pi M. Jiang C. J. Cheng B. Yu J. G. (2017). Synthesis of hierarchical porous zinc oxide (ZnO) microspheres with highly efficient adsorption of Congo red. J. Colloid Interrface Sci. 490, 242–251. 10.1016/j.jcis.2016.11.049 27912123

[B29] Li T. M. Zeng W. Wang Z. C. (2015). Quasi-one-dimensional metal-oxide-based heterostructural gas-sensing materials: a review. Sens. Actuators B Chem. 221, 1570–1585. 10.1016/j.snb.2015.08.003

[B30] Li W. Q. Ma S. Y. Yang G. J. Mao Y. Z. Luo J. Cheng L. (2015). Preparation, characterization and gas sensing properties of pure and Ce doped ZnO hollow nanofibers. Mater. Lett. 138, 188–191. 10.1016/j.matlet.2014.09.130

[B31] Li X. Li X. Wang J. Lin S. (2015). Highly sensitive and selective room-temperature formaldehyde sensors using hollow TiO_2_ microspheres, Sens. Actuators B Chem. 219, 158–163. 10.1016/j.snb.2015.05.031

[B32] Liao L. Mai H. X. Yuan Q. Lu H. B. Li J. C. Liu C. (2008). Single CeO_2_ nanowire gas sensor supported with Pt nanocrystals: gas sensitivity, surface bond states, and chemical mechanism. J. Phys. Chem. C 112, 9061–9065. 10.1021/jp7117778

[B33] Lin Y. Wei W. Wang Y. Zhou J. R. Sun D. M. Zhang X. D. (2015). Highly stabilized and rapid sensing acetone sensor based on Au nanoparticle-decorated flower-like ZnO microstructures. J. Alloy Compd. 650, 37–44. 10.1016/j.jallcom.2015.07.242

[B34] Liu C. Zhao L. P. Wang B. Q. Sun P. Wang Q. J. Gao Y.. (2017). Acetone gas sensor based on NiO/ZnO hollow spheres: Fast response and recovery, and low (ppb) detection limit. J. Colloid Interrface Sci. 495, 207–215. 10.1016/j.jcis.2017.01.106 28237094

[B35] Lu G. Y. Xu J. Sun J. B. Yu Y. S. Zhang Y. Q. Liu F. M. (2012). UV-enhanced room temperature NO_2_ sensor using ZnO nanorods modified with SnO_2_ nanoparticles. Sens. Actuators B Chem. 162, 82–88. 10.1016/j.snb.2011.12.039

[B36] Ma G. M. Li C. R. Luo Y. T. Mu R. D. Wang L. (2012). High sensitive and reliable fiber bragg grating hydrogen sensor for fault detection of power transformer. Sens. Actuators B Chem. 169, 195–198. 10.1016/j.snb.2012.04.066

[B37] Mariprasath T. Kirubakaran V. (2018). A real time study on condition monitoring of distribution transformer using thermal imager. Infrared Phys. Technol. 90, 78–86. 10.1016/j.infrared.2018.02.009

[B38] Mirzaei A. Kim J. H. Kim H. W. Kim S. S. (2018). How shell thickness can affect the gas sensing properties of nanostructured materials: Survey of literature. Sens. Actuators B Chem. 258, 270–294. 10.1016/j.snb.2017.11.066

[B39] Mo M. S. Lim S. H. Mai Y. W. Zheng R. K. Ringer S. P. (2008). *In situ* self-assembly of thin ZnO nanoplatelets into hierarchical mesocrystal microtubules with surface grafting of nanorods: a general strategy towards hollow mesocrystal structures. Adv. Mater. 20, 339–342. 10.1002/adma.200701137

[B40] Nakate U. T. Patil P. Bulakhe R. N. Lokhande C. D. Kale S. N. Naushad M.. (2016). Sprayed zinc oxide films: ultra-violet light-induced reversible surface wettability and platinum-sensitization-assisted improved liquefied petroleum gas response. J. Colloid Interface Sci. 480, 109–117. 10.1016/j.jcis.2016.07.010 27421113

[B41] Park S. An S. Mun Y. Lee C. (2013). UV-enhanced NO_2_ gas sensing properties of SnO_2_-core/ZnO-shell nanowire at room temperature. ACS Appl. Mater. Interf. 5, 4285–4292. 10.1021/am400500a 23627276

[B42] Rout C. S. Govindaraj A. Rao C. (2006). High-sensitivity hydrocarbon sensors based on tungsten oxide nanowires. J. Mater. Chem. 16, 3936–3941. 10.1039/B607012B

[B43] Seiyama T. Kato A. Fujiishi K. Nagatani M. (1962). A new detector for gaseous components using semiconductive thin films. Anal. Chem. 34, 1502–1503. 10.1021/ac60191a001

[B44] Shimizu Y. Egashira M. (1999). Basic aspects and challenges of semiconductor gas sensors. MRS Bull. 24, 18–24. 10.1557/S0883769400052465

[B45] Siada A. A. Hmood S. (2015). A new fuzzy logic approach to identify power transformer criticality using dissolved gas-in-oil analysis. Int. J. Electr. Power 67, 401–408. 10.1016/j.ijepes.2014.12.017

[B46] Song L. M. Yue H. Li H. Y. Liu L. Li Y. Du L. T. (2018). Hierarchical porous ZnO microflowers with ultra-high ethanol gas sensing at low concentration. Chem. Phys. Lett. 699, 1–7. 10.1016/j.cplett.2018.03.021

[B47] Sun R. Wang Z. C. Saito M. Shibata N. Ikuhara Y. (2015). Atomistic mechanisms of nonstoichiometry-induced twin boundary structural transformation in titanium dioxide. Nat. Commun., 6, 7120–7126. 10.1038/ncomms8120 25958793 PMC4432645

[B48] Sun Y. F. Liu S. B. Meng F. L. Liu J. Y. Jin Z. Kong L. T.. (2012). Metal oxide nanostructures and their gas sensing properties: a review. Sensors 12, 2610–2631. 10.3390/s120302610 22736968 PMC3376589

[B49] Uddin A. Yaqoob U. Chung G. S. (2016). Dissolved hydrogen gas analysis in transformer oil using Pd catalyst decorated on ZnO nanorod array. Sens. Actuators B Chem. 226, 90–95. 10.1016/j.snb.2015.11.110

[B50] Vallejos S. Stoycheva T. Umek P. Navio C. Snyders R. Bittencourt C.. (2011). Au nanoparticle-functionalised WO_3_ nanoneedles and their application in high sensitivity gas sensor devices. Chem. Commun. 47, 565–567. 10.1039/c0cc02398a 21103469

[B51] Wang H. Qu Y. Chen H. Lin Z. D. Dai K. (2014). Highly selective n-butanol gas sensor based on mesoporous SnO_2_ prepared with hydrothermal treatment. Sens. Actuators B Chem. 201, 153–159. 10.1016/j.snb.2014.04.049

[B52] Wang X. Liu W. Liu J. Wang F. Kong J. Qiu S.. (2012). Synthesis of nestlike ZnO hierarchically porous structures and analysis of their gas sensing properties. ACS Appl. Mater. Interfaces 4, 817–825. 10.1021/am201476b 22216881

[B53] Xu T. T. Xu Y. M. Zhang X. F. Deng Z. P. Huo L. H. Gao S. (2018). Enhanced H_2_S Gas-Sensing Performance of Zn_2_SnO_4_ Lamellar Micro-Spheres. Front. Chem. 6:165. 10.3389/fchem.2018.00165 29868566 PMC5960710

[B54] Yang D. J. Kamienchick I. Youn D. Y. Rothschild A. Kim I. D. (2010). Ultrasensitive and highly selective gas sensors based on electrospun SnO_2_ nanofibers modified by Pd loading. Adv. Funct. Mater. 20, 4258–4264. 10.1002/adfm.201001251

[B55] Yang F. Jung D. Penner R. M. (2011). Trace detection of dissolved hydrogen gas in oil using a palladium nanowire array. Anal. Chem. 83, 9472–9477. 10.1021/ac2021745 22017676

[B56] Zeng W. Zhu L. P. Zhang Z. Y. Ye Z. Z. (2015). Fabrication of gas sensor based on mesoporous rhombus-shaped ZnO rod arrays. Sens. Actuators B Chem. 208, 112–121. 10.1016/j.snb.2014.11.024

[B57] Zhang Y. Xu J. Q. Xiang Q. Li H. Pan Q. Y. Xu P. C. (2009). Brush-Like hierarchical ZnO nanostructures: synthesis, photoluminescence and gas sensor properties. J. Phys. Chem. C 113, 3430–3435. 10.1021/jp8092258

[B58] Zhao T. Nguyen N. T. Xie Y. Sun X. F. Li Q. Li X. (2017). Inorganic Nanocrystals Functionalized Mesoporous Silica Nanoparticles: Fabrication and Enhanced Bio-applications. Front. Chem. 5:118. 10.3389/fchem.2017.00118 29326923 PMC5733462

[B59] Zhu L. Zeng W. (2017). Room-temperature gas sensing of ZnO-based gas sensor: A review. Sens. Actuators A Phys. 267, 242–261. 10.1016/j.sna.2017.10.021

