# Supplementary material for: Gas Sensing Performances of ZnO Hierarchical Structures for Detecting Dissolved Gases in Transformer Oil: A Mini Review
Source: Front Chem. 2018 Oct 22;6:508. doi: 10.3389/fchem.2018.00508 (PMC6204760; doi:10.3389/fchem.2018.00508)
Supplement: Supplementary file 1 [file Table_1.docx]

***Supplementary Material***

**Gas Sensing Performances of ZnO Hierarchical Structures**

**for Detecting Dissolved Gases in Transformer**

**Oil: A Mini Review**

**He Zhang^*^, Wei-Gen Chen^*^, Yan-Qiong Li, Zi-Hao Song**

*** Correspondence:** Ms. He Zhang: sophy305410@163.com and Prof. Wei-Gen Chen: weigench@cqu.edu.cn

**Supplementary Figures:**


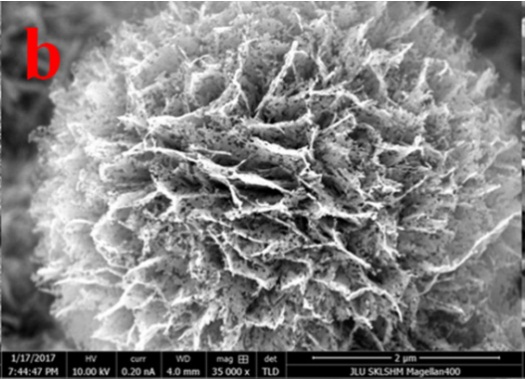


C

1 um


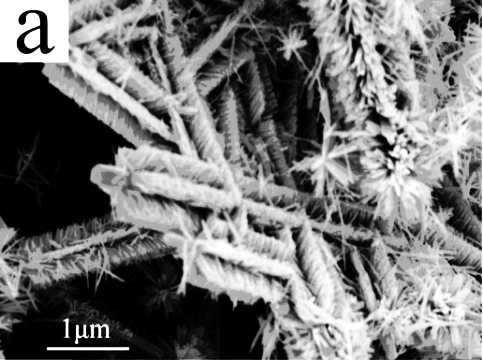


A

1um


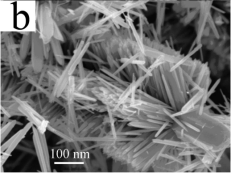


100 nm


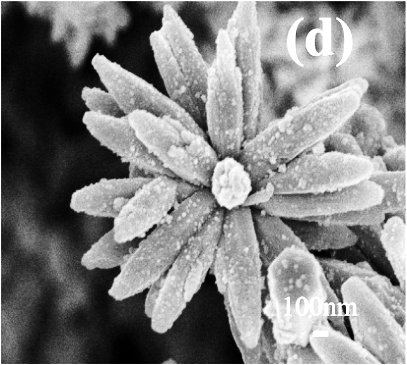


D

100 nm


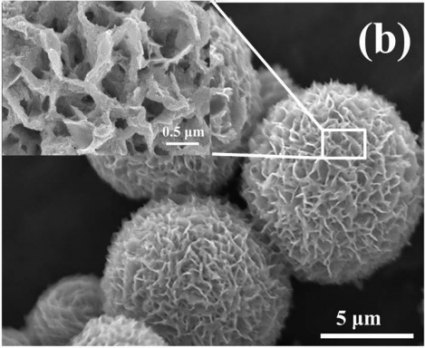


B

500 nm

5 um


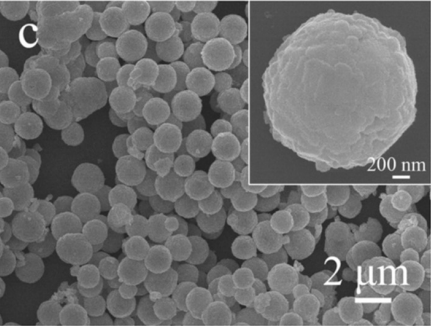


E

200 nm

2 um


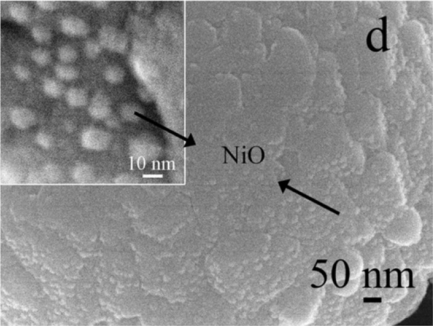


F

10 nm

50 nm


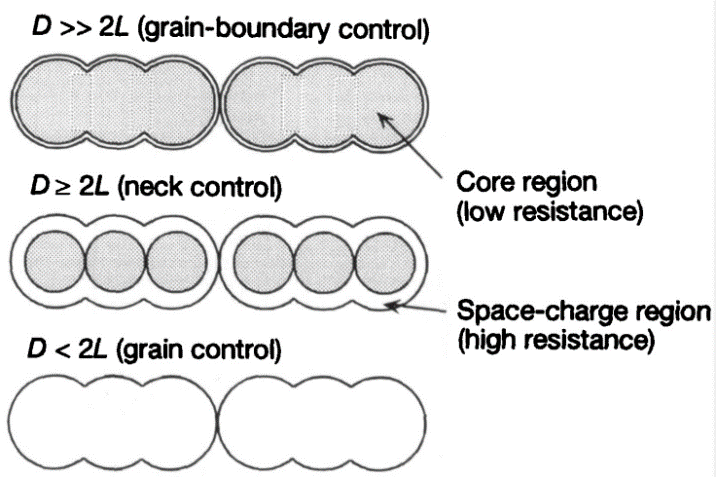


G

**Supplementary Figure 1.** FE-SEM images of the (a) brush-like hierarchical ZnO nanostructures. Reprinted with permission from Zhang et al. (2009). Copyright (2009) American Chemical Society. (b) porous ZnO. Reprinted with permission from Lei et al. (2017). Copyright (2017) Academic Press Inc Elsevier Science. (c) hierarchical porous ZnO microflowers. Reprinted with permission from Song et al. (2018). Copyright (2018) Elsevier Science BV. (d) Au nanoparticle-decorated flower-like ZnO microstructure. Reprinted with permission from Lin et al. (2015). Copyright (2015) Elsevier Science SA. (e) and (f) NiO/ZnO composites. Reprinted with permission from Liu et al. (2017). Copyright (2017) Academic Press Inc Elsevier Science. (g) three kinds of schematic models for grain-size effects. Reprinted with permission from Shimizu and Egashira, (1999). Copyright (1999) Materials Research Society.

**Response (Ra/Rg)**


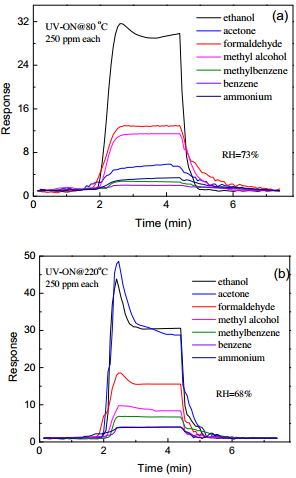


**Time (min)**

A

**Response (Ra/Rg)**


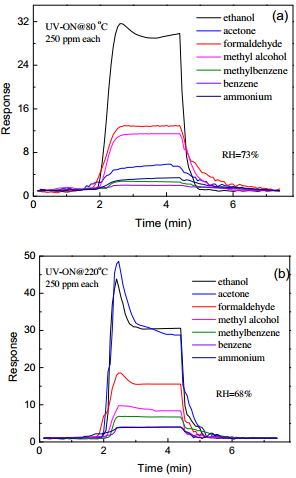


**Time (min)**

B


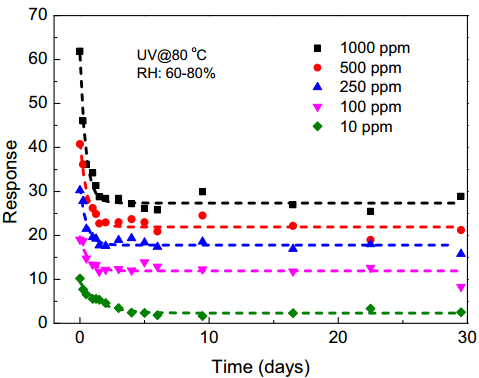


C

**Time (day)**

**Response (Ra/Rg)**

**Supplementary Figure 2.** The selectivity of the sensor to several possible interferents such as acetone, formaldehyde, methanol, benzene and methylbenzene with 250 ppm each at (a) 80°C with UV activation. (b) 220°C with UV activation. (c) the long-term stability of the sensor working at 80°C with UV activation. Reprinted with permission from Chen et al., (2016b). Copyright (2016) Elsevier Science SA.


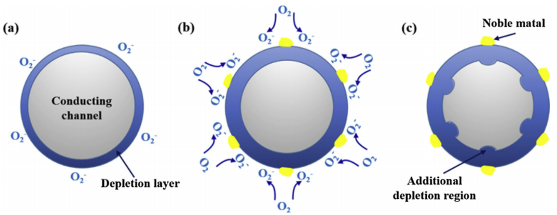


A

B

C

**Noble metal**

**Depletion layer**

**Additional depletion region**

**Supplementary Figure 3.** (a) Schematic diagram presenting depletion layer of unsensitized ZnO. Schematic illustrations of (b) chemical effect and (c) electric effect of noble metal sensitized ZnO. Reprinted with permission from Zhu and Zeng, (2017). Copyright (2017) Elsevier Science SA.
